# Virus Metagenomics in Farm Animals: A Systematic Review

**DOI:** 10.3390/v12010107

**Published:** 2020-01-16

**Authors:** Kirsty T. T. Kwok, David F. Nieuwenhuijse, My V. T. Phan, Marion P. G. Koopmans

**Affiliations:** Department of Viroscience, Erasmus MC, 3015 Rotterdam, The Netherlands; tt.kwok@erasmusmc.nl (K.T.T.K.); d.nieuwenhuijse@erasmusmc.nl (D.F.N.); v.t.m.phan@erasmusmc.nl (M.V.T.P.)

**Keywords:** virome, livestock, deep sequencing, animal reservoir, zoonosis, one health, viral metagenomics, NGS, emerging infectious diseases, high-throughput sequencing

## Abstract

A majority of emerging infectious diseases are of zoonotic origin. Metagenomic Next-Generation Sequencing (mNGS) has been employed to identify uncommon and novel infectious etiologies and characterize virus diversity in human, animal, and environmental samples. Here, we systematically reviewed studies that performed viral mNGS in common livestock (cattle, small ruminants, poultry, and pigs). We identified 2481 records and 120 records were ultimately included after a first and second screening. Pigs were the most frequently studied livestock and the virus diversity found in samples from poultry was the highest. Known animal viruses, zoonotic viruses, and novel viruses were reported in available literature, demonstrating the capacity of mNGS to identify both known and novel viruses. However, the coverage of metagenomic studies was patchy, with few data on the virome of small ruminants and respiratory virome of studied livestock. Essential metadata such as age of livestock and farm types were rarely mentioned in available literature, and only 10.8% of the datasets were publicly available. Developing a deeper understanding of livestock virome is crucial for detection of potential zoonotic and animal pathogens and One Health preparedness. Metagenomic studies can provide this background but only when combined with essential metadata and following the “FAIR” (Findable, Accessible, Interoperable, and Reusable) data principles.

## 1. Introduction

Emerging infectious diseases (EIDs) are responsible for a substantial burden of mortality and morbidity globally, and a majority of EIDs (60.3%) are caused by zoonotic pathogens [1,2,3]. In the past decades, high impact outbreaks have occurred with introductions from wildlife and livestock reservoirs, respectively, exemplified by the wildlife-borne outbreaks of Severe Acute Respiratory Syndrome (SARS) [4], Nipah disease [5,6], and Lassa fever [7], and outbreaks resulting from contact with livestock animals, such as Middle East Respiratory Syndrome [MERS] [8], avian influenza [9], Rift Valley fever [10], and swine influenza [11,12]. Livestock animals that live in close proximity to humans can facilitate transmission of infectious diseases through the wildlife-livestock-human interface [3].

Intensification of livestock farming is widely practiced in support of increasing food demand due to human population growth. This, in turn, facilitates disease transmissions within herds and between livestock and humans by increasing livestock population and density [3]. Therefore, the potential emergence of zoonoses from livestock population should not be underestimated [13,14]. In 2003, there was an outbreak of a highly pathogenic avian influenza A subtype H7N7 in humans linked to multiple commercial poultry farms in the Netherlands [15]. Two years later, another highly pathogenic avian influenza A H5N1 virus emerged in Asia in 2005, probably as a result of frequent mixing between flocks and wild birds, suggesting that pre-existing biosecurity measurements could not keep up with the rate of livestock intensification [16]. In 2007–2010, a large-scale Q fever outbreak was reported in the Netherlands, affecting more than 3500 human cases and resulting in a huge economic loss [17,18]. A steep increase in the number of goat farms most likely was the driver for the increased prevalence of *Coxiella burnetii* infections, with animal abortion waves that had gone unnoticed. The policy of voluntary reporting abortion outbreaks to the Animal Health Service hindered the timely detection of the circulation of Q fever, and therefore early interventions. These examples indicate that zoonotic risks in the livestock industry should be carefully managed and adapted to livestock intensification. The One Health approach has been coined for advocating collaboration between multiple stakeholders including veterinarians, clinicians, epidemiologists, virologists, microbiologists, ecologists, and policy makers to prevent and control EIDs through the human-animal-environment interface [19]. Surveillance of livestock and the surrounding environment is a hallmark of early detection but is currently targeted to known risks.

Advances in Next-Generation Sequencing (NGS) technologies and rapid development of bioinformatics and computational tools offer new opportunities for EID surveillance in quality and in scale. Particularly, metagenomic NGS (mNGS) allows unbiased detection of all microbes and viruses in a sample, showing potential for timely detection of rare or novel infectious etiologies, as well as for surveillance of foodborne and waterborne viruses [20,21,22,23,24,25,26]. However, the use of mNGS as a potential surveillance tool requires a deeper understanding of what is “normal” diversity in humans [27], as well as wildlife [28,29] and farm animals [30]. Characterizing species-specific metagenomes could potentially be used to provide a surveillance baseline for early detection and for tracking of movements of pathogens across different hosts, and has been promoted by projects like the global virome project [31]. However, for such applications, detailed background is needed regarding coverage, representativeness, and biases in the study designs. Here, we conduct a systematic review to identify available literature that performed viral mNGS in common farm animals including cattle, small ruminants (goats and sheep), poultry, and pigs. We reviewed the data and metadata availability and quality of these studies. We further summarized reported viromes of common farm animals in order to translate these efforts as background virus diversity profiles of common farm animals to guide preparedness of diseases at the livestock-human interface.

## 2. Materials and Methods

### 2.1. Search Strategy and Selection Criteria

To provide an overview of studies that performed viral mNGS in common livestock including cattle, small ruminants (goats and sheep), poultry, and pigs, we performed a systematic review in five electronic databases (Embase, Medline, Cochrane Central, Web of Science, and Google Scholar) on 21 February 2019 using search terms such as “metagenomic,” “farm animals,” “livestock,” “virome,” and “high-throughput sequencing.” The detailed search strategy is described in the Supplementary Materials. All identified references were imported to Mendeley (available at https://www.mendeley.com/). Duplicated references were removed. 

Titles and abstracts of all unique references were screened by two independent reviewers (K.T.T.K. and D.F.N.). After first screening, full texts of remaining articles were assessed for eligibility. Articles that were not written in English were excluded. We also excluded conference abstracts and articles that did not report any original data (i.e., review papers, editorial, and commentaries). Only studies that performed viral metagenomic sequencing in specimens from farm animals were included. We included studies that focused on cattle, small ruminants (goats and sheep), poultry, and pigs. The flow of the systematic review was adapted from the guidelines of the Preferred Reporting Items for Systematic Reviews and Meta-Analyses (PRISMA) [32].

### 2.2. Data Extraction and Analysis

We extracted the following information from each included study: first author, journal name, year of publication, type of farm animals, breed type, health condition of the animals, age of the animals, sampling date, sample size, specimen type, farm type, location of the farm, geographical reference of the farm, virus detected, virus family detected, nucleic acid extraction method, sequencing platform, Sequence Read Archive (SRA)/European Nucleotide Archive (ENA)/GenBank record (if any), and rationale of the study.

Data cleaning, stratification, analysis, and visualization was performed using R packages [33] (dplyr [34], ggplot2 [35], reshape2 [36], and stringr [37]). Information on host range and taxonomy of different virus families was adapted from ViralZone [38] and International Committee Taxonomy of Viruses [39]. The host range of different virus families were stratified into seven groups: vertebrate virus, invertebrate virus, bacteriophage, plant virus, other virus not classified into the first four categories (e.g., mycovirus and archaeal virus etc.), virus with multiple hosts, and unclassified virus/virus with unknown host(s). Geographical origins of included studies were stratified according to World Health Organization (WHO) regions: Africa region, the Americas, Eastern Mediterranean region, European region, South-East Asia region, and the Western Pacific region. Health conditions of farm animals were stratified into four major groups: healthy, gastrointestinal signs, respiratory signs, and other clinical signs. The category “other clinical signs” refers to any clinical signs that could not be classified into the first three categories and unspecified health conditions. Studies were stratified into seven arbitrary types based on their primary findings: virome study (i.e., papers that focused on studying the virus diversity of the animals), genetic characterization, mNGS as a diagnostic tool, mNGS as a diagnostic tool and virome study, virus discovery (i.e., papers that primarily focused on discovery of novel viruses), methodology papers, and others. Sequencing platforms were stratified into four main sequencing platforms: 454 pyrosequencing (Roche), Illumina, Ion Torrent (Thermo Fisher), and Oxford Nanopore Technologies. Sample sizes were stratified into six arbitrary groups: less than 10, 11–50, 51–100, 101–500, 501–1500, and unspecified sample size. Nucleic acid extraction strategies were stratified into four groups: column-based, solvent-based (e.g., chloroform and TRIzol [Thermo Fisher]), magnetic bead-based, and other/multiple types of extraction methods. 

## 3. Results

### 3.1. Overall Descriptions of All Studies

**Figure 1:**
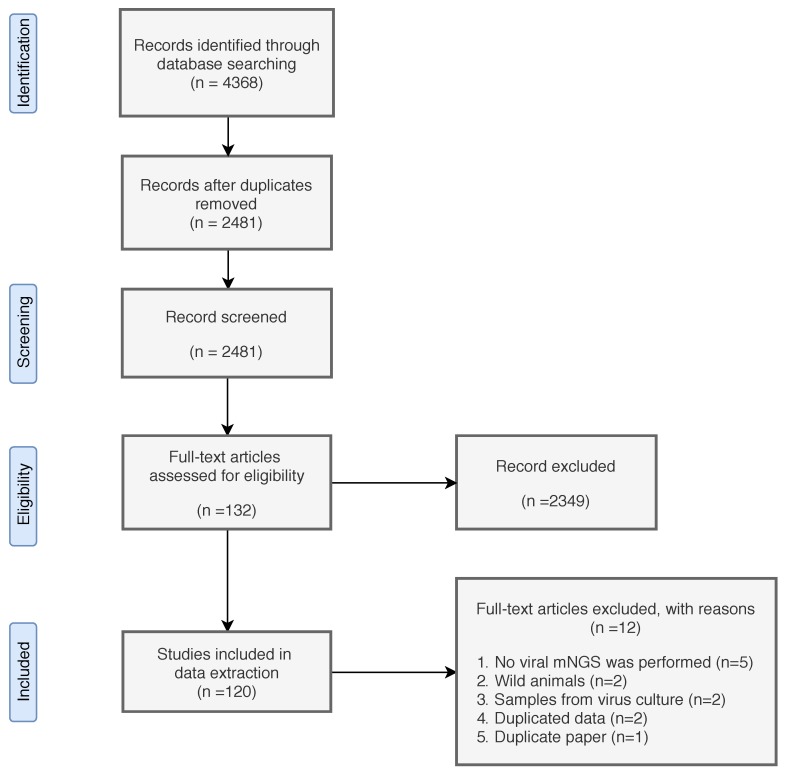
Flow chart of systematic review. The procedures were adapted from the Preferred Reporting Items for Systematic Reviews and Meta-Analyses (PRISMA) guidelines.

A total of 4368 records were identified (Figure 1). After record deduplication, we performed the first screening with 2481 included records. After first screening, 2349 records were excluded for the following reasons: no mNGS was performed, studied animals were wild animals or were not cattle, small ruminants (goat and sheep), poultry, and/or pigs, records were not written in English, records did not contain original findings, or records were conference abstracts. Full text of 132 remaining records were assessed for eligibility, and 120 records were included for data extraction (Supplementary Materials).

**Figure 2:**
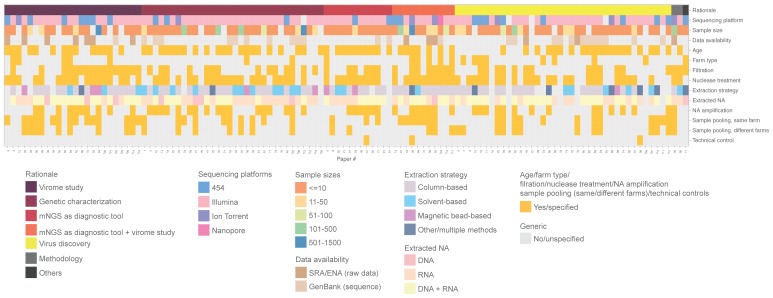
An overview of study design and data (and metadata) availability of 120 included publications. Each column represents one paper, and numbers refer to the list of references (Supplementary Materials). Rows describe data fields extracted from the papers as follows (from top to bottom): study rationale, sequencing platforms, sample sizes, availability of sequencing data in public repository, availability of animal age description, availability of farm type description, indication of whether filtration was performed, indication of whether nuclease treatment was performed during sample preparation, nucleic acid extraction strategy, type of nucleic acid extracted, indication of whether nucleic acid amplification was performed, indication of whether sample pooling was performed in same/different farms, and mention of technical controls for validating sequencing results. Categories and color codes for each data field are indicated in the figure legend. Gray boxes indicate that the technique was not performed, or the information was not specified. mNGS = metagenomic Next-Generation Sequencing. SRA = Sequence Read Archive. ENA = European Nucleotide Archive. NA = nucleic acid.

In terms of metadata availability, only one-fifth of the included studies specified farm types and nearly 40% of the studies did not mention the age of the farm animals (Figure 2). Twenty-eight percent and 40% of studies pooled specimens between farms and within farms together for sequencing, respectively. More than 95% of the studies did not specify whether there were technical controls for validating the sequencing results. For data availability, only 10.8% (N = 13) provided raw sequencing data in the public repository, either the SRA or ENA. There is no distinct pattern when comparing metadata and data availability in studies with different study rationales. 

**Figure 3:**
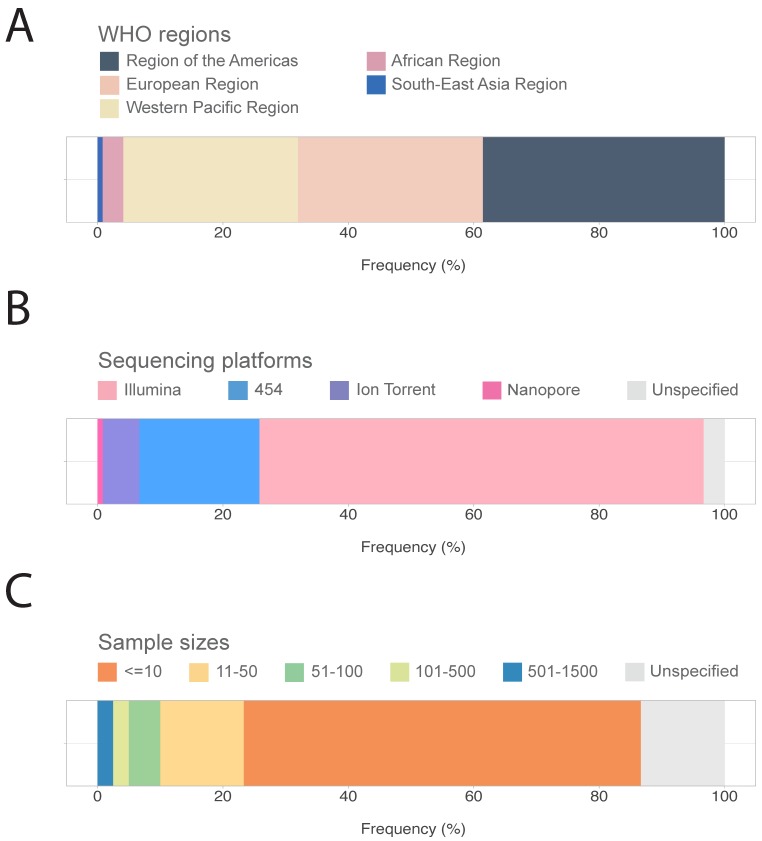
(**A**) Geographical origin of included studies stratified by World Health Organization (WHO) regions. (**B**) An overview of sequencing platforms. (**C**) An overview of sample sizes.

Geographically, most studies were carried out in three out of six WHO regions: the Americas (38.3%, N = 46), followed by European region (29.2%, N = 35), and the Western Pacific region (28.3%, N = 34) (Figure 3A). There were four studies in the African region (3.3%) and one study in the South-East Asia region (0.8%), and we did not identify publications from the Eastern Mediterranean region.

For sequencing platform, the majority of the studies (70.9%, N = 85) performed NGS with Illumina sequencing platform, followed by 454 pyrosequencing (19.2%, N = 23), and Ion Torrent sequencing (5.8%, N = 7). (Figure 2 and Figure 3B). Only one study sequenced with the Nanopore platform (0.8%). Four studies (3.3%) did not specify which sequencing platforms were used [40,41,42,43]. The earliest included study was published in 2009. 454 pyrosequencing was the most popular platform during 2009–2013, gradually replaced by Illumina starting from 2014 (Supplementary Materials Figure S1).

In terms of viral enrichment strategies, we found that half of the included studies performed filtration steps, majority (68.3%, N = 82) performed nuclease treatment, and 45.8% (N = 55) studies performed DNA/RNA amplification steps (Figure 2). Almost half of the studies (N = 58) extracted both DNA and RNA and around one-third of the studies extracted RNA only (N = 44). For extraction strategies, majority used the column-based method (N = 62), followed by the solvent-based method (19.2%, N = 23), and the magnetic bead-based method (6.7%, N = 8).

Over 75% of studies had a sample size of less than a 100 (Figure 2 and Figure 3C). Six studies (5%) and three studies (2.5%) had a sample size of 101–500 and 501–1500, respectively. Of note, the majority of the studies (63.3%, N = 76) had a sample size range <= 10. Sixteen studies (13.3%) did not report sample size.

### 3.2. Farm Animals, Health Conditions, and Specimen Types

Pigs were the most frequently studied animals (N = 64) among all four types of farm animals in the available literature, followed by cattle (N = 29), poultry (N = 19), and small ruminants (goats and sheep) (N = 9). Only one study provided data on more than one type of farm animals.

Most studies involving pigs and poultry included healthy animals (N = 26 for pigs and N = 6 for poultry). For cattle, there were seven studies each for healthy animals, animals with respiratory signs, and animals with gastrointestinal signs. For small ruminants, there were two studies each for healthy animals and animals with gastrointestinal signs; there was no mNGS study available for small ruminants that had respiratory signs.

**Figure 4:**
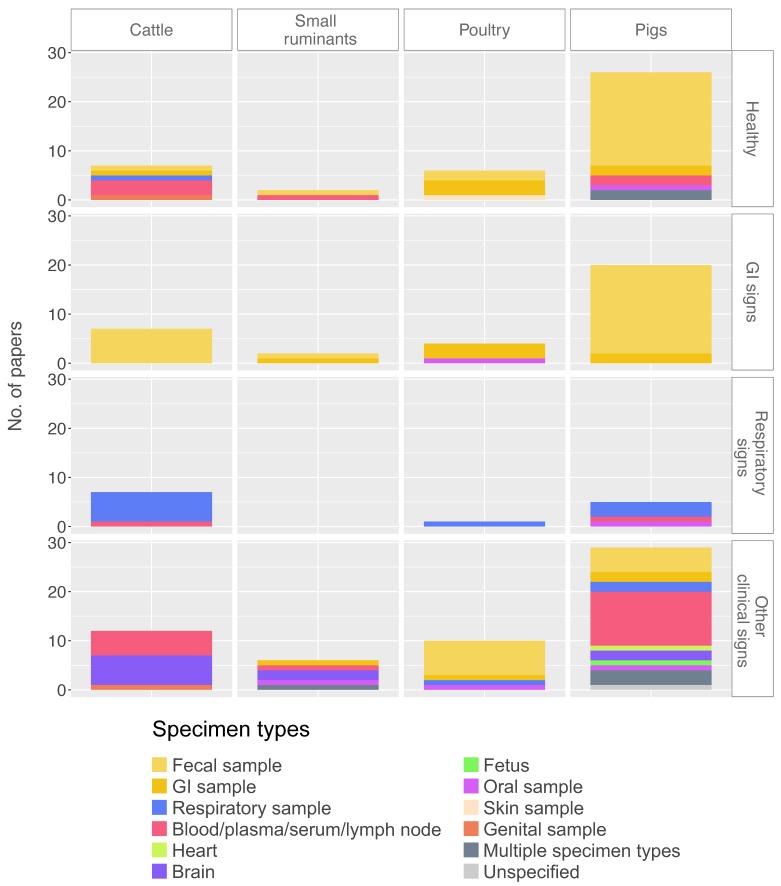
Types of specimens tested in different farm animals by number of papers, stratified by reported health conditions. Categories per variable are color-coded as shown in the legend. GI = gastrointestinal. A version of *Y*-axis as frequency (%) can be found in Supplementary Materials Figure S2.

Furthermore, we looked into specimens collected for the four major health conditions. For healthy farm animals, specimen types were slightly skewed towards gastrointestinal samples, particularly in pigs (81%) and poultry (83%) (Figure 4). Specimen type diversity was highest in both cattle and pigs (N = 5), followed by poultry (N = 3), and small ruminants (N = 2). For respiratory samples, there was only one study with healthy cattle [44], whereas no studies in healthy pigs, poultry, and small ruminants were identified (Figure 4). For symptomatic animals, the sampling strategy reflected the clinical signs (i.e., gastrointestinal sample for gastrointestinal signs, respiratory samples for respiratory signs) (Figure 4).

### 3.3. Virus Diversity in Different Farm Animals

**Figure 5:**
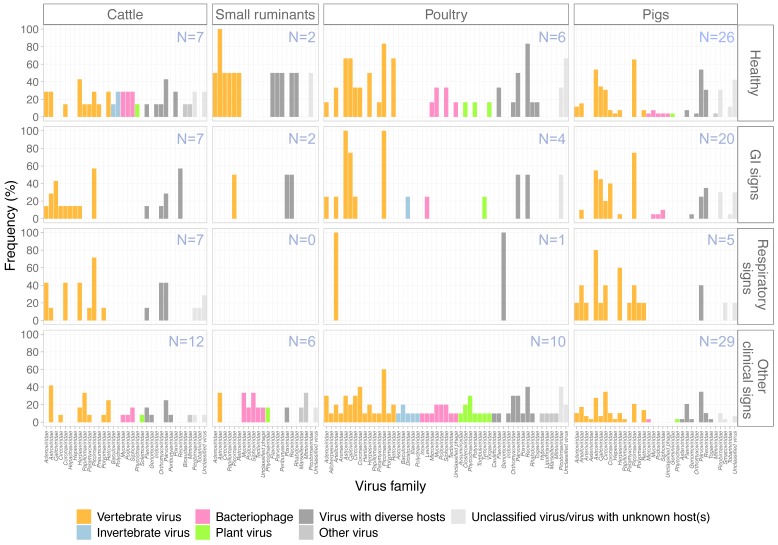
An overview of virus families found in different farm animals in included studies, stratified by reported health conditions. The bars are color-coded by host range of the viruses as shown in the figure legend. GI = gastrointestinal. *Y* axis shows proportion of studies that found the viruses specified.

**Figure 6:**
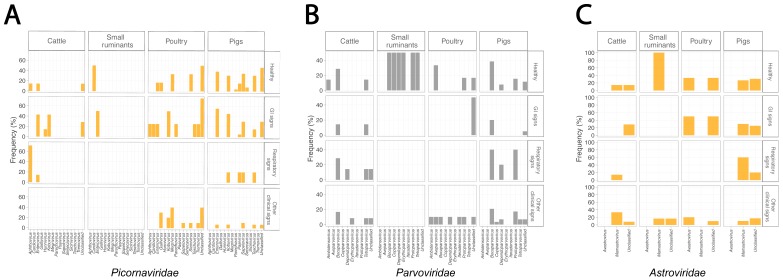
Frequencies of virus genera of top three most abundant virus families stratified by health conditions. (**A**) *Picornaviridae*. (**B**) *Parvoviridae*. (**C**) *Astroviridae*. Color code is based on host range and adapted from Figure 5.

The greatest diversity of viruses was observed in samples from poultry, with 49 virus families reported in the available literature, followed by cattle with viruses found from 33 families and pigs with 32 families (Figure 5). In comparison, only 20 virus families were reported in studies involving small ruminants. Fifteen vertebrate virus families were reported in both pigs and poultry, followed by cattle (N = 14) and small ruminants (N = 6). Apart from vertebrate viruses, bacteriophages and plant viruses were found in all four types of livestock while invertebrate viruses were only detected in cattle and poultry. Bacteriophage belonging to families *Myoviridae*, *Podoviridae*, and *Siphoviridae* were detected in all four livestock types. Interestingly, the diversity of plant viruses was identified highest in poultry with seven plant virus families detected (compared to two each for pigs and cattle, and one for small ruminants). Overall, *Picornaviridae*, *Parvoviridae*, and *Astroviridae* were among the three most frequently found virus families identified. There was no distinguishable pattern when comparing genera of the above-listed three most abundantly found virus families in the four major health conditions (Figure 6). *Enterovirus*, *bocaparvovirus*, and *dependoparvovirus* were reported in all four livestock types (Figure 6). Viruses within the *Astroviridae* family were host species-specific as *avastrovirus* and *mamastrovirus* were only found in poultry and mammals, respectively (Figure 6C).

*Reoviridae* and *Caliciviridae* were frequently reported in studies of healthy poultry (83% and 66%, respectively) and poultry with gastrointestinal (GI) signs (50% and 75%, respectively). *Herpesviridae* were reported in 60% of studies of pigs with respiratory signs. In general, the reported virome of healthy farm animals and farm animals with clinical signs were somewhat similar. A diverse range of known animal viruses (listed in Table 1) and newly recognized viruses (listed in Table 2) were reported in mNGS studies. Notably, some viruses that are known to have zoonotic potential were reported in available mNGS studies including hepatitis E viruses in gastrointestinal samples of pigs [45,46] and cattle [47] and Influenza A viruses in respiratory samples of poultry [25] and pigs [46]. 

## 4. Discussion

The rapid and extensive development of NGS has opened up more opportunities to advance understanding in infectious disease diagnostics, surveillance, and transmission [133]. One of the key NGS applications is the primer-independent, agnostic (i.e., without prior knowledge) viral metagenomics to characterize all viruses present in the samples, and also allow the discovery of novel or uncommon infectious etiologies [134,135]. In this review, we summarize previous studies that performed viral mNGS in common farm animals including cattle, small ruminants, poultry, and pigs in an effort to provide background virus diversity profiles of these farm animals. Information systematically summarized from this review will help to guide the design of future studies employing mNGS for surveillance as well as preparedness for detection of diseases at the livestock-human interface.

The majority of available farm animal viral mNGS literature studied pigs, which may be explained by the emergence and global spread of swine viruses such as porcine epidemic diarrhea virus [136] and African Swine Fever virus [137] in the recent decade. Indeed, these viruses were reported in our included studies [75,88]. Other mNGS studies also found hepatitis E viruses in pigs, known to be zoonotic. In addition to these known animal viruses, a wide range of other viruses have been described in different livestock by using mNGS, highlighting the potential for using mNGS to identify not only viruses that affect the animals, but also zoonotic and novel viruses.

Although the global population of goats and sheep exceeds that of pigs [138], only nine mNGS studies of samples from small ruminants were identified in our review and hence it is not surprising that reported virus diversity of small ruminants was the lowest when compared to other common farm animals. Even though zoonoses from small ruminants such as chlamydiosis are thought to be transmitted via direct contact, the example of Q fever has shown the potential for spread by inhalation of contaminated aerosol [139]. The largest Dutch Q-fever outbreak in 2007–2010 with more than 3500 cases certainly indicated that zoonotic risk from small ruminants should not be underestimated [18], and in line with the predominance of viruses as causes of emerging disease outbreaks, studies are needed to characterize the virome of small ruminants and its possible relationship to the health and disease of exposed humans.

The viromes of farm animals in health and disease in the reviewed mNGS studies were relatively comparable. It could be that some infections were subclinical, for instance, porcine noroviruses and rotaviruses have been found in asymptomatic pigs as well as diarrheic pigs [140,141]. Also, as the major clinical syndromes can be caused by a range of pathogens, it is unlikely that a single predominant virus would be found in diseased animals, unless it would be a highly prevalent disease cause. This highlights the challenge in incorporating complex viral metagenomic data in disease association studies. In addition, disease association studies require deeper taxonomic annotation of virus sequences to the level of genus and species, which can be challenging with short read data provided by the most commonly used sequencing platforms, although sequence assembly methods have been improved considerably [142]. 

We showed that *Picornaviridae*, *Parvoviridae*, and *Astroviridae* were among the most commonly identified virus families in common farm animals. Therefore, although sample sizes were relatively small, these virus families may be signature viruses to indicate livestock exposure. For instance, *Avastrovirus*, an avian virus, so far has only been reported in poultry sample metagenomes. Multiple signature viruses would be required to set up a viral fingerprint profile for each livestock type, as one genetic marker may not be sensitive and/or specific enough. In our studies, we aim to identify stable signatures to allow tracking the flow of viruses between different livestock, humans, and the surrounding environment. 

There are limitations in our review dataset of the available farm animal mNGS studies. Reporting bias might be introduced with different study objectives and research interests, the use of different algorithms for metagenomic analysis, reference databases [143], and different sample preparation strategies (e.g., presence/absence of nuclease treatment, filtration and random amplification, different sizes of filters used, and centrifugation strategies) [144]. Some studies performed pooling among samples from different farms. This practice is cost-effective for a resource-limited setting, however viruses with low abundance may be diluted and missed. About 90% of studies did not provide raw sequencing data on SRA or ENA which hampers cross-study comparison and future large-scale reanalysis and interpretation. This is crucial, as it has been shown that outputs from metagenomic workflows are not directly comparable with the use of different algorithms and reference databases. Data sharing is also warranted for successful pathogen surveillance and outbreak detection [145,146] and our review shows that there is much to be gained in the field of metagenomics. It was difficult to compare virome composition of farm animals from different farm types and of different ages in available studies. Over 80% and 40% of available studies did not specify farm type and age of farm animals. We observed that metadata availability is consistently lacking in available literature regardless of different study types. Our review shows that the current state of the art is far from providing such information as the minimal metadata that is needed to select datasets for analysis is rarely provided with published reports. In the longer term, a better understanding of the association between farm types and virome composition of farm animals would guide farm management practices for reducing risks from zoonoses. In the meantime, it is important to benchmark metagenomic tools and reference databases [147,148] and assess epidemic potential of pathogens for more accurate zoonoses prediction and detection [149].

Available viral mNGS studies of healthy farm animals primarily focused on fecal virome, probably because fecal samples of farm animals are a non-invasive sample and are readily available to be collected. However, one must notice that recently EIDs are like MERS, such as the avian and swine influenza, and are mostly respiratory related, although viral shedding of MERS coronavirus was also reported in camel and human stools and some MERS patients also experienced diarrhea [150]. Nonetheless, viral mNGS studies of respiratory samples of farm animals are substantially overlooked and underreported. 

To our best knowledge, this is the first systematic review that summarizes available literature that performed viral mNGS in farm animal samples. These aforementioned limitations of the reviewed dataset may hinder the reusability of the published metagenomic data. An incredible amount of metagenomics data is being generated these days as we speak, given reduced costs of sequencing and increased availability of NGS platforms. Therefore, timing is perfect to take one step further to implement good data management practices referred as the “FAIR” (Findable, Accessible, Interoperable, and Reusable) data principles [151]. In brief, data should be properly indexed and easy to find and assess. Data should also be described with rich and well-defined metadata that can be interpreted by humans and computers. By making metagenomic data “FAIR”, reusability of the data can be optimized so the data can be combined into meta-analysis settings. Minimum datasets for the sharing of pathogen genomic data have already been established through the global microbial identifier project [152]. We propose to always include breed types, age of the animals, and farm types in the minimal metadata set to allow determination of viral fingerprints of different farm animals. More “FAIR” metagenomic data of farm animals would be favorable for future large-scale farm animal virome characterization analysis that can guide zoonotic outbreak preparedness.

This review provides an overview of available literature that performed viral mNGS in common livestock including cattle, small ruminants, poultry, and pigs. We summarized virus diversity of these farm animals reported in available literature and reviewed their study designs. This is a good starting point for identifying species signatures for porcine, bovine, and poultry fecal viromes, but major gaps in the data of the virus diversity of small ruminants exist. Given the lack and inconsistency of data and metadata availability in available literature, it is important to apply the “FAIR” data principles in future farm animal mNGS studies or any other related studies for enhancing data reusability. In the longer term, developing a better understanding of farm animal virome is crucial for detection of potential zoonotic pathogens, zoonotic outbreak response, and preparedness [153], as well as the preparedness to combat livestock diseases.

## Figures and Tables

**Table 1:** Examples of known animal viruses reported in available metagenomic Next-Generation (mNGS) studies.

Farm Animal Type	Known Animal Viruses Found by mNGS Studies	References
Cattle	Bovine adenovirus	[44,48,49,50]
	Bovine coronavirus	[44,50,51,52]
	Bovine papillomavirus	[53,54]
	Bovine parvovirus	[44,48,50,52,53,54,55,56]
	Bovine respiratory syncytial virus	[44]
	Bovine rhinitis A virus	[44,48,49]
	Bovine rhinitis B virus	[44,48,49,52,57]
	Bovine viral diarrhoea virus	[44,47]
	Enterovirus	[44,47,50,58]
	Hepatitis E virus	[47]
	Herpesvirus	[44,48,50,52,54,58]
	Influenza D virus	[48,59]
	Kobuvirus	[47]
	Norovirus	[58,60]
	Rotavirus	[47,58,61]
Small ruminants	Enterovirus	[62]
	Orf virus	[63,64]
	Rotavirus	[64,65]
Poultry	Avastrovirus	[66,67,68,69,70]
	Aveparvovirus	[69]
	Chicken anaemia virus	[68]
	Gallivirus	[70,71]
	Gammacoronavirus	[69,70]
	Influenza A virus	[25]
	Megrivirus	[69,70,71,72,73]
	Rotavirus	[67,70,72,74]
	Sicinivirus	[71,72]
	Tremovirus	[68,70]
Pigs	African swine fever virus	[75]
	Bocaparvovirus	[24,51,76,77,78,79,80,81]
	Enterovirus	[46,76,77,78,82,83,84,85,86,87,88,89,90,91,92,93]
	Hepatitis E virus	[45,46]
	Influenza A virus	[46]
	Kobuvirus	[46,76,77,78,79,80,84,87,88,91,92,94,95]
	Porcine adenovirus	[46,94]
	Porcine circovirus	[24,30,46,79,94,95,96,97,98]
	Porcine cytomegalovirus	[94,95,99]
	Porcine epidemic diarrhea virus	[46,79,84,88,92,100]
	Porcine reproductive and respiratory syndrome virus	[95,101,102]
	Porcine respiratory coronavirus	[46]
	Rotavirus	[24,80,82,83,84,87,90,92,100,103,104,105,106]
	Sapelovirus	[30,76,77,78,79,80,82,84,87,88,90,92,93,94]
	Sapovirus	[30,76,77,78,84,87,88,90,92,93,107]
	Torque teno virus	[24,30,46,79,94,95,108]

**Table 2:** Examples of novel viruses found in farm animals by available mNGS studies.

Farm Animal Type	Novel Viruses Found by mNGS Studies	References
Cattle	Astrovirus	[109]
	CRESS-DNA virus	[50]
	Hepacivirus	[110]
	Nidovirus	[52]
	Papillomavirus	[111]
	Parvovirus	[112]
	Picornavirus	[113]
	Orthobunyavirus	[114]
	Rotavirus	[115]
Small ruminants	Astrovirus	[116,117]
Poultry	Coronavirus	[118]
	Picobirnavirus	[42,119]
	Picornavirus	[120,121]
Pigs	Astrovirus	[122,123]
	Bocavirus/Bocavirus-like	[98,122,124]
	Bufavirus	[125]
	Circovirus/Circovirus-like	[40,104,126]
	Enterovirus/Enterovirus-like	[85,89,92,93]
	Immunodeficiency-associated stool virus	[127]
	Ljungan-like viruses	[122]
	Parvovirus	[128,129]
	Porcine stool-associated circular virus	[82]
	Posavirus	[100]
	Pestivirus	[41,130]
	Picornavirus	[131]
	Picornavirales	[132]
	Teschovirus/Teschovirus-like	[90]

## Supplementary Materials

Supplementary materials are available at https://www.mdpi.com/1999-4915/12/1/107/s1.
