# Supplementary material for: Virus Metagenomics in Farm Animals: A Systematic Review
Source: Viruses. 2020 Jan 16;12(1):107. doi: 10.3390/v12010107 (PMC7019290; doi:10.3390/v12010107)
Supplement: Supplementary file 1 [file viruses-12-00107-s001.pdf]

viruses

Supplementary Materials

Supplementary Figure S1

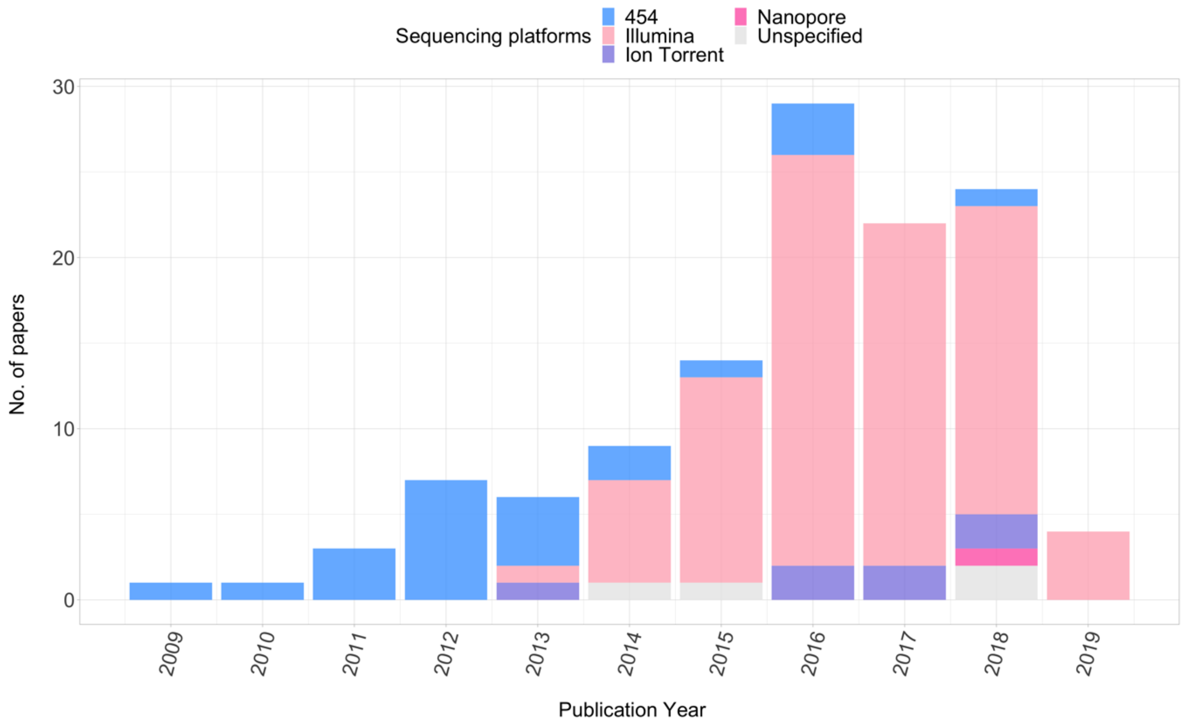

Supplementary Figure S1. The use of different Next-Generation Sequencing platforms in livestock viral metagenomics studies conducted during 2009-2019. X-axis shows the publication year of included studies. Y-axis shows number of papers. Bars are color-coded by the use of sequencing platforms as shown in the figure legend.

Supplementary Figure S2

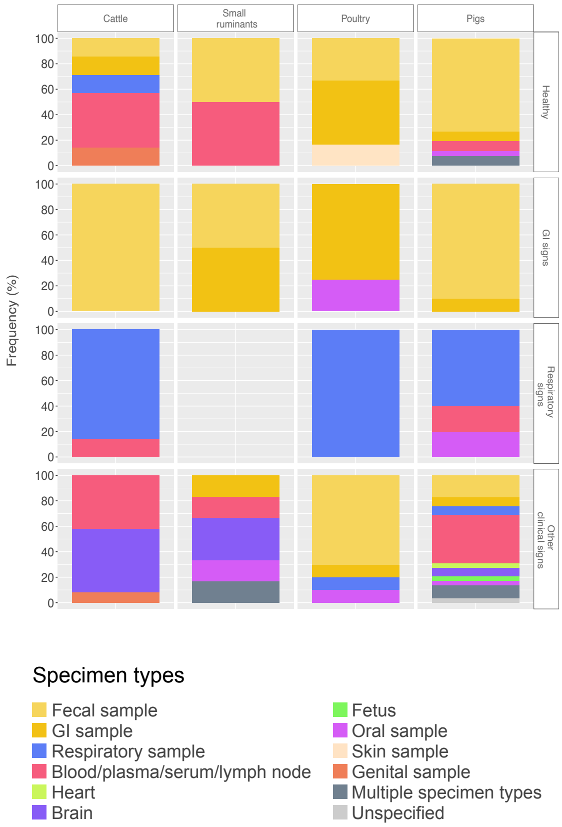

Supplementary Figure S2. Types of specimens tested in different farm animals by frequency (%), stratified by reported health conditions. Categories per variable are color coded as shown in the legend. GI = gastrointestinal.

Search strategies

Embase.com (Embase incl. Medline): 1806

('virome'/de OR 'virus classification'/de OR (('metagenomics'/de OR 'metagenome'/de OR 'high throughput sequencing'/de OR (metagenom* OR ((high-throughput OR shotgun) NEAR/3 (sequenc*))):ab,ti,kw) AND ('virus'/exp OR 'virology'/de OR 'virus infection'/exp OR (virus OR viral* OR virolog*):ab,ti,kw)) OR (virome* OR ((virus* OR viral*) NEAR/3 (classif* OR taxonom* OR phylogen*))):ab,ti,kw) AND ('livestock'/exp OR 'pig'/exp OR 'bovine'/exp OR 'goat'/exp OR 'sheep'/exp OR 'ruminant'/de OR 'cattle disease'/exp OR 'sheep disease'/exp OR 'swine disease'/exp OR 'foot and mouth disease'/de OR 'turkey (bird)'/exp OR 'duck'/exp OR 'bird disease'/exp OR (Livestock* OR ((farm* OR domestic*) NEAR/3 (animal*)) OR cattle* OR Cow* OR calve* OR bovine OR ovine OR porcine OR goat* OR sheep* OR lamb* OR ruminant* OR 'foot-and-mouth' OR 'hoof-and-mouth' OR poultry OR broiler* OR chicken* OR duck* OR pig OR pigs OR piglet* OR swine* OR boar OR hog):ab,ti) NOT ('Conference Abstract' OR Editorial)/it

Medline (Ovid): 1384

(((Metagenomics/ OR Metagenome/ OR High-Throughput Nucleotide Sequencing/ OR (metagenom* OR ((high-throughput OR shotgun) ADJ3 (sequenc*))).ab,ti,kw,kf.) AND (exp Viruses/ OR Virology/ OR exp Virus Diseases/ OR (virus OR viral* OR virolog*).ab,ti,kw,kf.)) OR (virome* OR ((virus* OR viral*) ADJ3 (classif* OR taxonom* OR phylogen*))).ab,ti,kw,kf.) AND (exp Animals, Domestic/ OR exp Swine/ OR Cattle/ OR exp Goats/ OR exp Sheep/ OR Ruminants/ OR exp Cattle Diseases/ OR exp Sheep Diseases/ OR exp Swine Diseases/ OR Goat Diseases/ OR Foot-and-Mouth Disease/ OR Ducks/ OR exp Bird Diseases/ OR (Livestock* OR ((farm* OR domestic*) ADJ3 (animal*)) OR cattle* OR Cow* OR calve* OR bovine OR ovine OR porcine OR piglet* OR goat* OR sheep* OR lamb* OR ruminant* OR foot-and-mouth OR hoof-and-mouth OR poultry OR broiler* OR chicken* OR duck* OR pig OR pigs OR piglet* OR swine* OR boar OR hog).ab,ti.) NOT (congresses OR editorial).pt.

Cochrane Central (trials): 1

((((metagenom* OR ((high-throughput OR shotgun) NEAR/3 (sequenc*))):ab,ti) AND ((virus OR viral* OR virolog*):ab,ti)) OR (virome* OR ((virus* OR viral*) NEAR/3 (classif* OR taxonom* OR phylogen*))):ab,ti) AND ((Livestock* OR ((farm* OR domestic*) NEAR/3 (animal*)) OR cattle* OR Cow* OR calve* OR bovine OR ovine OR porcine OR goat* OR sheep* OR lamb* OR ruminant* OR 'foot-and-mouth' OR 'hoof-and-mouth' OR poultry OR broiler* OR chicken* OR duck* OR pig OR pigs OR piglet* OR swine* OR boar OR hog):ab,ti)

Web of Science: 977

TS=(((("metagenomics"/de OR "metagenome"/de OR "high throughput sequencing"/de OR (metagenom* OR ((high-throughput OR shotgun) NEAR/2 (sequenc*)))) AND ((virus OR viral* OR virolog*))) OR (virome* OR ((virus* OR viral*) NEAR/2 (classif* OR taxonom* OR phylogen*)))) AND ((Livestock* OR ((farm* OR domestic*) NEAR/2 (animal*)) OR cattle* OR Cow* OR calve* OR bovine OR ovine OR porcine OR piglet* OR goat* OR sheep* OR lamb* OR ruminant* OR "foot-and-mouth" OR "hoof-and-mouth" OR poultry OR broiler* OR chicken* OR duck* OR pig OR pigs OR piglet* OR swine* OR boar OR hog))) AND DT=Article

Google Scholar: 200 (top relevant refs)

"metagenomic|high-throughput-sequencing|shotgun virus|viral|virology"|virome|"virus|viral classification|phylogeny" Livestock|"farm|domestic animal"|cattle|Cow|pig|piglet|swine|goat|sheep|poultry|chicken

References of 120 included studies

1. Akagami, M.; Ito, M.; Niira, K.; Kuroda, M.; Masuda, T.; Haga, K.; Tsuchiaka, S.; Naoi, Y.; Kishimoto, M.; Sano, K.; et al. Complete genome analysis of porcine kobuviruses from the feces of pigs in Japan. *Virus Genes* **2017**, *53*, 593–602.

2. Alekseev, K.P.; Penin, A.A.; Mukhin, A.N.; Khametova, K.M.; Grebennikova, T. V.; Yuzhakov, A.G.; Moskvina, A.S.; Musienko, M.I.; Raev, S.A.; Mishin, A.M.; et al. Genome characterization of a pathogenic porcine rotavirus B strain identified in Buryat republic, Russia in 2015. *Pathogens* **2018**, *7*.

3. Allen, H.K.; Looft, T.; Bayles, D.O.; Humphrey, S.; Levine, U.Y.; Alt, D.; Stanton, T.B. Antibiotics in feed induce prophages in swine fecal microbiomes. *MBio* **2011**, *2*.

4. Amimo, J.O.; El Zowalaty, M.E.; Githae, D.; Wamalwa, M.; Djikeng, A.; Nasrallah, G.K. Metagenomic analysis demonstrates the diversity of the fecal virome in asymptomatic pigs in East Africa. *Arch. Virol.* **2016**, *161*, 887–897.

5. Arruda, B.L.; Arruda, P.H.; Magstadt, D.R.; Schwartz, K.J.; Dohlman, T.; Schleining, J.A.; Patterson, A.R.; Visek, C.A.; Victoria, J.G. Identification of a divergent lineage porcine pestivirus in nursing piglets with congenital tremors and reproduction of disease following experimental inoculation. *PLoS One* **2016**, *11*, e0150104.

6. Arruda, B.; Arruda, P.; Hensch, M.; Chen, Q.; Zheng, Y.; Yang, C.; Gatto, I.R.H.; Ferreyra, F.M.; Gauger, P.; Schwartz, K.; et al. Porcine astrovirus type 3 in central nervous system of swine with polioencephalomyelitis. *Emerg. Infect. Dis.* **2017**, *23*, 2097–2100.

7. Baechlein, C.; Fischer, N.; Grundhoff, A.; Alawi, M.; Indenbirken, D.; Postel, A.; Baron, A.L.; Offinger, J.; Becker, K.; Beineke, A.; et al. Identification of a Novel Hepacivirus in Domestic Cattle from Germany. *J. Virol.* **2015**, *89*, 7007–7015.

8. Baechlein, C.; Grundhoff, A.; Fischer, N.; Alawi, M.; Hoeltig, D.; Waldmann, K.H.; Becher, P. Pegivirus infection in domestic pigs, Germany. *Emerg. Infect. Dis.* **2016**, *22*, 1312–1314.

9. Bauermann, F. V.; Joshi, L.R.; Mohr, K.A.; Kutish, G.F.; Meier, P.; Chase, C.; Christopher-Hennings, J.; Diel, D.G. A novel bovine papillomavirus type in the genus Dyokappapapillomavirus. *Arch. Virol.* **2017**, *162*, 3225–3228.

10. Bellehumeur, C.; Boyle, B.; Mandeville, I.; Gagnon, C.A. High-throughput sequencing revealed the presence of an unforeseen parvovirus species in Canadian swine: The porcine partetravirus. *Can. Vet. J.* **2013**, *54*, 787–789.

11. Berg Miller, M.E.; Yeoman, C.J.; Chia, N.; Tringe, S.G.; Angly, F.E.; Edwards, R.A.; Flint, H.J.; Lamed, R.; Bayer, E.A.; White, B.A. Phage-bacteria relationships and CRISPR elements revealed by a metagenomic survey of the rumen microbiome. *Environ. Microbiol.* **2012**, *14*, 207–227.

12. Blomström, A.L.; Belák, S.; Fossum, C.; McKillen, J.; Allan, G.; Wallgren, P.; Berg, M. Detection of a novel porcine boca-like virus in the background of porcine circovirus type 2 induced postweaning multisystemic wasting syndrome. *Virus Res.* **2009**, *146*, 125–129.

13. Blomström, A.L.; Fossum, C.; Wallgren, P.; Berg, M. Viral metagenomic analysis displays the Co-infection situation in healthy and PMWS affected pigs. *PLoS One* **2016**, *11*, e0166863.

14. Blomström, A.L.; Oma, V.; Khatri, M.; Hansen, H.H.; Stokstad, M.; Berg, M.; Myrmel, M. Genome sequence of a bovine rhinitis B virus identified in cattle in Sweden. *Genome Announc.* **2017**, *5*, e00172-17.

15. Blomström, A.L.; Ye, X.; Fossum, C.; Wallgren, P.; Berg, M. Characterisation of the virome of tonsils from conventional pigs and from specific pathogen-free pigs. *Viruses* **2018**, *10*, 382.

16. Boros, A.; Pankovics, P.; Knowles, N.J.; Nemes, C.; Delwart, E.; Reuter, G. Comparative Complete Genome Analysis of Chicken and Turkey Megriviruses (Family Picornaviridae): Long 3′ Untranslated Regions with a Potential Second Open Reading Frame and Evidence for Possible Recombination. *J. Virol.* **2014**, *88*, 6434–6443.

17. Boros, Á.; Nemes, C.; Pankovics, P.; Kapusinszky, B.; Delwart, E.; Reuter, G. Genetic characterization of a novel picornavirus in turkeys (Meleagris gallopavo) distinct from turkey galliviruses and megriviruses and distantly related to the members of the genus Avihepatovirus. *J. Gen. Virol.* **2013**, *94*, 1496–1509.

18. Boros, Á.; Nemes, C.; Pankovics, P.; Kapusinszky, B.; Delwart, E.; Reuter, G. Identification and complete genome characterization of a novel picornavirus in Turkey (Meleagris gallopavo). *J. Gen. Virol.* **2012**, *93*, 2171–2182.

19. Boros, Á.; Polgár, B.; Pankovics, P.; Fenyvesi, H.; Engelmann, P.; Phan, T.G.; Delwart, E.; Reuter, G. Multiple divergent picobirnaviruses with functional prokaryotic Shine-Dalgarno ribosome binding sites present in cloacal sample of a diarrheic chicken. *Virology* **2018**, *525*, 62–72.

20. Boujon, C.L.; Koch, M.C.; Wüthrich, D.; Werder, S.; Jakupovic, D.; Bruggmann, R.; Seuberlich, T. Indication of cross-species transmission of astrovirus associated with encephalitis in sheep and cattle. *Emerg. Infect. Dis.* **2017**, *23*, 1604–1606.

21. Bouzalas, I.G.; Wüthrich, D.; Selimovic-Hamza, S.; Drögemüller, C.; Bruggmann, R.; Seuberlich, T. Full-genome based molecular characterization of encephalitis-associated bovine astroviruses. *Infect. Genet. Evol.* **2016**, *44*, 162–168.

22. Bouzalas, I.G.; Wüthrich, D.; Walland, J.; Drögemüller, C.; Zurbriggen, A.; Vandevelde, M.; Oevermann, A.; Bruggmann, R.; Seuberlich, T. Neurotropic astrovirus in cattle with nonsuppurative encephalitis in Europe. *J. Clin. Microbiol.* **2014**, *52*, 3318–3324.

23. Campos, F.S.; Kluge, M.; Franco, A.C.; Giongo, A.; Valdez, F.P.; Saddi, T.M.; Brito, W.M.E.D.; Roehe, P.M. Complete genome sequence of porcine parvovirus 2 recovered from swine sera. *Genome Announc.* **2016**, *4*, e01627–15.

24. Chen, F.; Knutson, T.P.; Ciarlet, M.; Sturos, M.; Marthaler, D.G. Complete genome characterization of a rotavirus B (RVB) strain identified in alpine goat kids with enteritis reveals inter-species transmission with RVB bovine strains. *J. Gen. Virol.* **2018**, *99*, 457–463.

25. Chen, G.Q.; Zhuang, Q.Y.; Wang, K.C.; Liu, S.; Shao, J.Z.; Jiang, W.M.; Hou, G.Y.; Li, J.P.; Yu, J.M.; Li, Y.P.; et al. Identification and Survey of a Novel Avian Coronavirus in Ducks. *PLoS One* **2013**, *8*, e72918.

26. Chen, Q.; Wang, L.; Zheng, Y.; Zhang, J.; Guo, B.; Yoon, K.J.; Gauger, P.C.; Harmon, K.M.; Main, R.G.; Li, G. Metagenomic analysis of the RNA fraction of the fecal virome indicates high diversity in pigs infected by porcine endemic diarrhea virus in the United States. *Virol. J.* **2018**, *15*, 95.

27. Chen, X.; Zhang, B.; Yue, H.; Wang, Y.; Zhou, F.; Zhang, Q.; Tang, C. A novel astrovirus species in the gut of yaks with diarrhoea in the Qinghai-Tibetan plateau, 2013. *J. Gen. Virol.* **2015**, *96*, 3672–3680.

28. Chen, Z.; Collin, E.; Peddireddi, L.; Clement, T.; Gauger, P.; Hause, B.M. Genetic diversity in envelope genes of contemporary U.S. porcine reproductive and respiratory syndrome virus strains influences viral antigenicity. *Res. Vet. Sci.* **2017**, *115*, 432–441.

29. Cheung, A.K.; Ng, T.F.; Lager, K.M.; Bayles, D.O.; Alt, D.P.; Delwart, E.L.; Pogranichniy, R.M.; Kehrli, M.E. A divergent clade of circular single-stranded DNA viruses from pig feces. *Arch. Virol.* **2013**, *158*, 2157–2162.

30. Cheung, A.K.; Ng, T.F.F.; Lager, K.M.; Alt, D.P.; Delwart, E.L.; Pogranichniy, R.M. Unique circovirus-like genome detected in pig feces. *Genome Announc.* **2014**, *2*, e00251–14.

31. Cibulski, S.P.; Teixeira, T.F.; dos Santos, H.F.; de Sales Lima, F.E.; Scheffer, C.M.; Varela, A.P.M.; de Lima, D.A.; Schmidt, C.; Silveira, F.; de Almeida, L.L.; et al. Ungulate copiparvovirus 1 (bovine parvovirus 2):

characterization of a new genotype and associated viremia in different bovine age groups. *Virus Genes* **2016**, *52*, 134–137.

32. Conceição-Neto, N.; Theuns, S.; Cui, T.; Zeller, M.; Yinda, C.K.; Christiaens, I.; Heylen, E.; Van Ranst, M.; Carpentier, S.; Nauwynck, H.J.; et al. Identification of an enterovirus recombinant with a torovirus-like gene insertion during a diarrhea outbreak in fattening pigs. *Virus Evol.* **2017**, *3*, vex024.

33. Day, J.M.; Ballard, L.L.; Duke, M. V.; Scheffler, B.E.; Zsak, L. Metagenomic analysis of the turkey gut RNA virus community. *Virol. J.* **2010**, *7*, 313.

34. Day, J.M.; Zsak, L. Investigating Turkey Enteric Picornavirus and Its Association with Enteric Disease in Poults. *Avian Dis.* **2015**, *59*, 138–142.

35. De Souza, W.M.; Dennis, T.; Fumagalli, M.J.; Araujo, J.; Sabino-Santos, G.; Maia, F.G.M.; Acrani, G.O.; Carrasco, A.D.O.T.; Romeiro, M.F.; Modha, S.; et al. Novel parvoviruses from wild and domestic animals in Brazil provide new insights into parvovirus distribution and diversity. *Viruses* **2018**, *10*, 143.

36. Denesvre, C.; Dumarest, M.; Rémy, S.; Gourichon, D.; Eloit, M. Chicken skin virome analyzed by high-throughput sequencing shows a composition highly different from human skin. *Virus Genes* **2015**, *51*, 209–216.

37. Devaney, R.; Trudgett, J.; Trudgett, A.; Meharg, C.; Smyth, V. A metagenomic comparison of endemic viruses from broiler chickens with runting-stunting syndrome and from normal birds. *Avian Pathol.* **2016**, *45*, 616–629.

38. Dumarest, M.; Muth, E.; Cheval, J.; Gratigny, M.; Hébert, C.; Gagnieur, L.; Eloit, M. Viral diversity in swine intestinal mucus used for the manufacture of heparin as analyzed by high-throughput sequencing. *Biologicals* **2015**, *43*, 31–36.

39. Escalera-Zamudio, M.; Taboada, B.; Rojas-Anaya, E.; Löber, U.; Loza-Rubio, E.; Arias, C.F.; Greenwood, A.D. Viral Communities Among Sympatric Vampire Bats and Cattle. *Ecohealth* **2018**, *15*, 132–142.

40. Farlow, J.; Donduashvili, M.; Kokhreidze, M.; Kotorashvili, A.; Vepkhvadze, N.G.; Kotaria, N.; Gulbani, A. Intra-epidemic genome variation in highly pathogenic African swine fever virus (ASFV) from the country of Georgia. *Virol. J.* **2018**, *15*, 190.

41. Ferragut, F.; Vega, C.G.; Mauroy, A.; Conceição-Neto, N.; Zeller, M.; Heylen, E.; Uriarte, E.L.; Bilbao, G.; Bok, M.; Matthijnssens, J.; et al. Molecular detection of bovine Noroviruses in Argentinean dairy calves: Circulation of a tentative new genotype. *Infect. Genet. Evol.* **2016**, *40*, 144–150.

42. Grierson, S.S.; McGowan, S.; Cook, C.; Steinbach, F.; Choudhury, B. Molecular and in vitro characterisation of hepatitis E virus from UK pigs. *Virology* **2019**, *527*, 116–121.

43. Guo, Z.; He, Q.; Tang, C.; Zhang, B.; Yue, H. Identification and genomic characterization of a novel CRESS DNA virus from a calf with severe hemorrhagic enteritis in China. *Virus Res.* **2018**, *255*, 141–146.

44. Hanke, D.; Pohlmann, A.; Sauter-Louis, C.; Höper, D.; Stadler, J.; Ritzmann, M.; Steinrigl, A.; Schwarz, B.A.; Akimkin, V.; Fux, R.; et al. Porcine epidemic diarrhea in europe: In-detail analyses of disease dynamics and molecular epidemiology. *Viruses* **2017**, *9*, 177.

45. Hargitai, R.; Pankovics, P.; Kertész, A.M.; Bíró, H.; Boros, Á.; Phan, T.G.; Delwart, E.; Reuter, G. Detection and genetic characterization of a novel parvovirus distantly related to human bufavirus in domestic pigs. *Arch. Virol.* **2016**, *161*, 1033–1037.

46. Hause, B.M.; Duff, J.W.; Scheidt, A.; Anderson, G. Virus detection using metagenomic sequencing of swine nasal and rectal swabs. *J. Swine. Heal. Prod.* **2016**, *24*, 304–308.

47. Hause, B.M.; Collin, E.A.; Anderson, J.; Hesse, R.A.; Anderson, G. Bovine rhinitis viruses are common in U.S. cattle with bovine respiratory disease. *PLoS One* **2015**, *10*, e0121998.

48. Hause, B.M.; Collin, E.A.; Peddireddi, L.; Yuan, F.; Chen, Z.; Hesse, R.A.; Gauger, P.C.; Clement, T.; Fang, Y.; Anderson, G. Discovery of a novel putative atypical porcine pestivirus in pigs in the USA. *J. Gen. Virol.* **2015**, *96*, 2994–2998.

49. Hause, B.M.; Hesse, R.A.; Anderson, G.A. Identification of a novel Picornavirales virus distantly related to posavirus in swine feces. *Virus Genes* **2015**, *51*, 144–147.

50. Hause, B.M.; Myers, O.; Duff, J.; Hesse, R.A. Senecavirus A in pigs, United States, 2015. *Emerg. Infect. Dis.* **2016**, *22*, 1323–1325.

51. Hause, B.M.; Padmanabhan, A.; Pedersen, K.; Gidlewski, T. Feral swine virome is dominated by single-stranded DNA viruses and contains a novel Orthopneumovirus which circulates both in feral and domestic swine. *J. Gen. Virol.* **2016**, *97*, 2090–2095.

52. Hause, B.M.; Smith, C.; Bishop, B.; Stewart, C.; Simonson, R. Complete genome sequence of a porcine polyomavirus from nasal swabs of pigs with respiratory disease. *Genome Announc.* **2018**, *6*, e00344-18.

53. Hayashi-Miyamoto, M.; Murakami, T.; Minami-Fukuda, F.; Tsuchiaka, S.; Kishimoto, M.; Sano, K.; Naoi, Y.; Asano, K.; Ichimaru, T.; Haga, K.; et al. Diversity in VP3, NSP3, and NSP4 of rotavirus B detected from Japanese cattle. *Infect. Genet. Evol.* **2017**, *49*, 97–103.

54. Hoffmann, B.; Scheuch, M.; Höper, D.; Jungblut, R.; Holsteg, M.; Schirrmeier, H.; Eschbaumer, M.; Goller, K. V.; Wernike, K.; Fischer, M.; et al. Novel orthobunyavirus in cattle, Europe, 2011. *Emerg. Infect. Dis.* **2012**, *18*, 469–472.

55. Ito, M.; Kuroda, M.; Masuda, T.; Akagami, M.; Haga, K.; Tsuchiaka, S.; Kishimoto, M.; Naoi, Y.; Sano, K.; Omatsu, T.; et al. Whole genome analysis of porcine astroviruses detected in Japanese pigs reveals genetic diversity and possible intra-genotypic recombination. *Infect. Genet. Evol.* **2017**, *50*, 38–48.

56. Karlsson, O.E.; Larsson, J.; Hayer, J.; Berg, M.; Jacobson, M. The intestinal eukaryotic virome in healthy and diarrhoeic neonatal piglets. *PLoS One* **2016**, *11*, e0151481.

57. Kim, H.R.; Yoon, S.J.; Lee, H.S.; Kwon, Y.K. Identification of a picornavirus from chickens with transmissible viral proventriculitis using metagenomic analysis. *Arch. Virol.* **2015**, *160*, 701–709.

58. Knutson, T.P.; Velayudhan, B.T.; Marthaler, D.G. A porcine enterovirus G associated with enteric disease contains a novel papain-like cysteine protease. *J. Gen. Virol.* **2017**, *98*, 1305–1310.

59. Kuroda, M.; Masuda, T.; Ito, M.; Naoi, Y.; Doan, Y.H.; Haga, K.; Tsuchiaka, S.; Kishimoto, M.; Sano, K.; Omatsu, T.; et al. Genetic diversity and intergenogroup recombination events of sapoviruses detected from feces of pigs in Japan. *Infect. Genet. Evol.* **2017**, *55*, 209–217.

60. Lager, K.M.; Ng, T.F.; Bayles, D.O.; Alt, D.P.; Delwart, E.L.; Cheung, A.K. Diversity of viruses detected by deep sequencing in pigs from a common background. *J. Vet. Diagnostic Investig.* **2012**, *24*, 1177–1179.

61. Li, J.; Zhang, W.; Cui, L.; Shen, Q.; Hua, X. Metagenomic identification, genetic characterization and genotyping of porcine sapoviruses. *Infect. Genet. Evol.* **2018**, *62*, 244–252.

62. Li, L.; Diab, S.; McGraw, S.; Barr, B.; Traslavina, R.; Higgins, R.; Talbot, T.; Blanchard, P.; Rimoldi, G.; Fahsbender, E.; et al. Divergent astrovirus associated with neurologic disease in cattle. *Emerg. Infect. Dis.* **2013**, *19*, 1385–1392.

63. Lima, D.A.; Cibulski, S.P.; Finkler, F.; Teixeira, T.F.; Varela, A.P.M.; Cerva, C.; Loiko, M.R.; Scheffer, C.M.; Dos Santos, H.F.; Mayer, F.Q.; et al. Faecal virome of healthy chickens reveals a large diversity of the eukaryote viral community, including novel circular ssDNA viruses. *J. Gen. Virol.* **2017**, *98*, 690–703.

64. Lima, D.A.; Cibulski, S.P.; Tochetto, C.; Varela, A.P.M.; Finkler, F.; Teixeira, T.F.; Loiko, M.R.; Cerva, C.; Junqueira, D.M.; Mayer, F.Q.; et al. The intestinal virome of malabsorption syndrome-affected and unaffected broilers through shotgun metagenomics. *Virus Res.* **2019**, *261*, 9–20.

65. Ling, Y.; Zhang, X.; Qi, G.; Yang, S.; Jingjiao, L.; Shen, Q.; Wang, X.; Cui, L.; Hua, X.; Deng, X.; et al. Viral metagenomics reveals significant viruses in the genital tract of apparently healthy dairy cows. *Arch. Virol.* **2019**, *164*, 1059–1067.

66. Liu, L.; Schwarz, L.; Ullman, K.; Ahola, H.; Qiu, Y.; Ma, Z.; Hennig-Pauka, I. Identification of a novel bufavirus in domestic pigs by a viral metagenomic approach. *J. Gen. Virol.* **2016**, *97*, 1592–1596.

67. Maganga, G.D.; Relmy, A.; Bakkali-Kassimi, L.; Ngoubangoye, B.; Tsoumbou, T.; Bouchier, C.; N'Dilimabaka, N.; Leroy, E.M.; Zientara, S.; Berthet, N. Molecular characterization of Orf virus in goats in Gabon, Central Africa. *Virol. J.* **2016**, *13*, 79.

68. Masembe, C.; Michuki, G.; Onyango, M.; Rumberia, C.; Norling, M.; Bishop, R.P.; Djikeng, A.; Kemp, S.J.; Orth, A.; Skilton, R.A.; et al. Viral metagenomics demonstrates that domestic pigs are a potential reservoir for Ndumu virus. *Virol. J.* **2012**, *9*, 218.

69. Masuda, T.; Nagai, M.; Yamasato, H.; Tsuchiaka, S.; Okazaki, S.; Katayama, Y.; Oba, M.; Nishiura, N.; Sassa, Y.; Omatsu, T.; et al. Identification of novel bovine group A rotavirus G15P[14] strain from epizootic diarrhea of adult cows by de novo sequencing using a next-generation sequencer. *Vet. Microbiol.* **2014**, *171*, 66–73.

70. Masuda, T.; Sunaga, F.; Naoi, Y.; Ito, M.; Takagi, H.; Katayama, Y.; Omatsu, T.; Oba, M.; Sakaguchi, S.; Furuya, T.; et al. Whole genome analysis of a novel picornavirus related to the Enterovirus/Sapelovirus supergroup from porcine feces in Japan. *Virus Res.* **2018**, *257*, 68–73.

71. Mekata, H.; Yamamoto, M.; Hamabe, S.; Tanaka, H.; Omatsu, T.; Mizutani, T.; Hause, B.M.; Okabayashi, T. Molecular epidemiological survey and phylogenetic analysis of bovine influenza D virus in Japan. *Transbound. Emerg. Dis.* **2018**, *65*, e355–e360.

72. Mitra, N.; Cernicchiaro, N.; Torres, S.; Li, F.; Hause, B.M. Metagenomic characterization of the virome associated with bovine respiratory disease in feedlot cattle identified novel viruses and suggests an etiologic role for influenza D virus. *J. Gen. Virol.* **2016**, *97*, 1771–1784.

73. Nagai, M.; Omatsu, T.; Aoki, H.; Kaku, Y.; Belsham, G.J.; Haga, K.; Naoi, Y.; Sano, K.; Umetsu, M.; Shiokawa, M.; et al. Identification and complete genome analysis of a novel bovine picornavirus in Japan. *Virus Res.* **2015**, *210*, 205–212.

74. Nagai, M.; Omatsu, T.; Aoki, H.; Otomaru, K.; Uto, T.; Koizumi, M.; Minami-Fukuda, F.; Takai, H.; Murakami, T.; Masuda, T.; et al. Full genome analysis of bovine astrovirus from fecal samples of cattle in Japan: identification of possible interspecies transmission of bovine astrovirus. *Arch. Virol.* **2015**, *160*, 2491–2501.

75. Namonyo, S.; Wagacha, M.; Maina, S.; Wambua, L.; Agaba, M. A metagenomic study of the rumen virome in domestic caprids. *Arch. Virol.* **2018**, *163*, 3415–3419.

76. Naoi, Y.; Kishimoto, M.; Masuda, T.; Ito, M.; Tsuchiaka, S.; Sano, K.; Yamasato, H.; Omatsu, T.; Aoki, H.; Furuya, T.; et al. Characterization and phylogenetic analysis of a novel picornavirus from swine feces in Japan. *Arch. Virol.* **2016**, *161*, 1685–1690.

77. Ng, T.F.F.; Kondov, N.O.; Deng, X.; Van Eenennaam, A.; Neibergs, H.L.; Delwart, E. A metagenomics and case-control study to identify viruses associated with bovine respiratory disease. *J. Virol.* **2015**, *89*, 5340–5349.

78. Niira, K.; Ito, M.; Masuda, T.; Saitou, T.; Abe, T.; Komoto, S.; Sato, M.; Yamasato, H.; Kishimoto, M.; Naoi, Y.; et al. Whole genome sequences of Japanese porcine species C rotaviruses reveal a high diversity of genotypes of individual genes and will contribute to a comprehensive, generally accepted classification system. *Infect. Genet. Evol.* **2016**, *44*, 106–113.

79. Oba, M.; Katayama, Y.; Tsuchiaka, S.; Omatsu, T.; Murata, Y.; Ohya, K.; Makino, S.; Nagai, M.; Mizutani, T. Discovery of genome of an immunodeficiency-associated virus-like virus from pig feces in Japan. *Jpn. J. Vet. Res.* **2018**, *66*, 53–56.

80. Oba, M.; Naoi, Y.; Ito, M.; Masuda, T.; Katayama, Y.; Sakaguchi, S.; Omatsu, T.; Furuya, T.; Yamasato, H.; Sunaga, F.; et al. Metagenomic identification and sequence analysis of a Teschovirus A-related virus in porcine feces in Japan, 2014–2016. *Infect. Genet. Evol.* **2018**, *66*, 210–216.

81. Omatsu, T.; Tsuchiaka, S.; Hirata, T.; Shiroma, Y.; Okazaki, S.; Katayama, Y.; Oba, M.; Nishiura, N.; Sassa, Y.; Furuya, T.; et al. Detection of enterovirus genome sequence from diarrheal feces of goat. *Virus Genes* **2014**, *48*, 550–552.

82. Padmanabhan, A.; Hause, B.M. Detection and characterization of a novel genotype of porcine astrovirus 4 from nasal swabs from pigs with acute respiratory disease. *Arch. Virol.* **2016**, *161*, 2575–2579.

83. Palinski, R.M.; Mitra, N.; Hause, B.M. Discovery of a novel Parvovirinae virus, porcine parvovirus 7, by metagenomic sequencing of porcine rectal swabs. *Virus Genes* **2016**, *52*, 564–567.

84. Palinski, R.; Piñeyro, P.; Shang, P.; Yuan, F.; Guo, R.; Fang, Y.; Byers, E.; Hause, B.M. A novel porcine circovirus distantly related to known circoviruses is associated with porcine dermatitis and nephropathy syndrome and reproductive failure. *J. Virol.* **2017**, *91*, e01879-16.

85. Pankovics, P.; Boros, Á.; Nemes, C.; Kapusinszky, B.; Delwart, E.; Reuter, G. Molecular characterization of a novel picobirnavirus in a chicken. *Arch. Virol.* **2018**, *163*, 3455–3458.

86. Pfaff, F.; Schlottau, K.; Scholes, S.; Courtenay, A.; Hoffmann, B.; Höper, D.; Beer, M. A novel astrovirus associated with encephalitis and ganglionitis in domestic sheep. *Transbound. Emerg. Dis.* **2017**, *64*, 677–682.

87. Pfankuche, V.M.; Bodewes, R.; Hahn, K.; Puff, C.; Beineke, A.; Habierski, A.; Osterhaus, A.D.M.E.; Baumgärtner, W. Porcine bocavirus infection associated with encephalomyelitis in a pig, Germany. *Emerg. Infect. Dis.* **2016**, *22*, 1310–1312.

88. Phan, T.G.; Giannitti, F.; Rossow, S.; Marthaler, D.; Knutson, T.; Li, L.; Deng, X.; Resende, T.; Vannucci, F.; Delwart, E. Detection of a novel circovirus PCV3 in pigs with cardiac and multi-systemic inflammation. *Virol. J.* **2016**, *13*, 1–8.

89. Qin, S.; Ruan, W.; Yue, H.; Tang, C.; Zhou, K.; Zhang, B. Viral communities associated with porcine respiratory disease complex in intensive commercial farms in Sichuan province, China. *Sci. Rep.* **2018**, *8*, 13341.

90. Qiu, Y.; Chen, J.M.; Wang, T.; Hou, G.Y.; Zhuang, Q.Y.; Wu, R.; Wang, K.C. Detection of viromes of RNA viruses using the next generation sequencing libraries prepared by three methods. *Virus Res.* **2017**, *237*, 22–26.

91. Reuter, G.; Pankovics, P.; Delwart, E.; Boros, Á. Identification of a novel astrovirus in domestic sheep in Hungary. *Arch. Virol.* **2012**, *157*, 323–327.

92. Rosseel, T.; Scheuch, M.; Höper, D.; de Regge, N.; Caij, A.B.; Vandenbussche, F.; van Borm, S. DNase SISPA-next generation sequencing confirms Schmallenberg virus in Belgian field samples and identifies genetic variation in Europe. *PLoS One* **2012**, *7*, e41967.

93. Sachsenröder, J.; Twardziok, S.O.; Scheuch, M.; Johne, R. The general composition of the faecal virome of pigs depends on age, but not on feeding with a probiotic bacterium. *PLoS One* **2014**, *9*, e88888.

94. Sadeghi, M.; Kapusinszky, B.; Yugo, D.M.; Phan, T.G.; Deng, X.; Kanevsky, I.; Opriessnig, T.; Woolums, A.R.; Hurley, D.J.; Meng, X.J.; et al. Virome of US bovine calf serum. *Biologicals* **2017**, *46*, 64–67.

95. Sajnani, M.R.; Sudarsanam, D.; Pandit, R.J.; Oza, T.; Hinsu, A.T.; Jakhesara, S.J.; Solosanc, S.; Joshi, C.G.; Bhatt, V.D. Metagenomic data of DNA viruses of poultry affected with respiratory tract infection. *Data Br.* **2018**, *16*, 157–160.

96. Sano, K.; Naoi, Y.; Kishimoto, M.; Masuda, T.; Tanabe, H.; Ito, M.; Niira, K.; Haga, K.; Asano, K.; Tsuchiaka, S.; et al. Identification of further diversity among posaviruses. *Arch. Virol.* **2016**, *161*, 3541–3548.

97. Schirtzinger, E.E.; Suddith, A.W.; Hause, B.M.; Hesse, R.A. First identification of porcine parvovirus 6 in North America by viral metagenomic sequencing of serum from pigs infected with porcine reproductive and respiratory syndrome virus.. *Virol. J.* **2015**, *12*, 170.

98. Schlottau, K.; Schulze, C.; Bilk, S.; Hanke, D.; Höper, D.; Beer, M.; Hoffmann, B. Detection of a Novel Bovine Astrovirus in a Cow with Encephalitis. *Transbound. Emerg. Dis.* **2016**, *63*, 253–259.

99. Shah, J.D.; Desai, P.T.; Zhang, Y.; Scharber, S.K.; Baller, J.; Xing, Z.S.; Cardona, C.J. Development of the Intestinal RNA Virus Community of Healthy Broiler Chickens. *PLoS One* **2016**, *11*, e0150094.

100. Shah, J.D.; Baller, J.; Zhang, Y.; Silverstein, K.; Xing, Z.; Cardona, C.J. Comparison of tissue sample processing methods for harvesting the viral metagenome and a snapshot of the RNA viral community in a turkey gut. *J. Virol. Methods* **2014**, *209*, 15–24.

101. Shan, T.; Li, L.; Simmonds, P.; Wang, C.; Moeser, A.; Delwart, E. The Fecal Virome of Pigs on a High-Density Farm. *J. Virol.* **2011**, *85*, 11697–11708.

102. Steel, O.; Kraberger, S.; Sikorski, A.; Young, L.M.; Catchpole, R.J.; Stevens, A.J.; Ladley, J.J.; Coray, D.S.; Stainton, D.; Dayaram, A.; et al. Circular replication-associated protein encoding DNA viruses identified in the faecal matter of various animals in New Zealand. *Infect. Genet. Evol.* **2016**, *43*, 151–164.

103. Theuns, S.; Conceição-Neto, N.; Zeller, M.; Heylen, E.; Roukaerts, I.D.M.; Desmarets, L.M.B.; Van Ranst, M.; Nauwynck, H.J.; Matthijnssens, J. Characterization of a genetically heterogeneous porcine rotavirus C, and other viruses present in the fecal virome of a non-diarrheic Belgian piglet. *Infect. Genet. Evol.* **2016**, *43*, 135–145.

104. Theuns, S.; Vanmechelen, B.; Bernaert, Q.; Deboutte, W.; Vandenhole, M.; Beller, L.; Matthijnssens, J.; Maes, P.; Nauwynck, H.J. Nanopore sequencing as a revolutionary diagnostic tool for porcine viral enteric disease complexes identifies porcine kobuvirus as an important enteric virus. *Sci. Rep.* **2018**, *8*, 9830.

105. Tochetto, C.; Lima, D.A.; Varela, A.P.M.; Loiko, M.R.; Paim, W.P.; Scheffer, C.M.; Herpich, J.I.; Cerva, C.; Schmitd, C.; Cibulski, S.P.; et al. Full-Genome Sequence of Porcine Circovirus type 3 recovered from serum of sows with stillbirths in Brazil. *Transbound. Emerg. Dis.* **2018**, *65*, 5–9.

106. Tokarz, R.; Sameroff, S.; Hesse, R.A.; Hause, B.M.; Desai, A.; Jain, K.; Ian Lipkin, W. Discovery of a novel nidovirus in cattle with respiratory disease. *J. Gen. Virol.* **2015**, *96*, 2188–2193.

107. Tsuchiaka, S.; Naoi, Y.; Imai, R.; Masuda, T.; Ito, M.; Akagami, M.; Ouchi, Y.; Ishii, K.; Sakaguchi, S.; Omatsu, T.; et al. Genetic diversity and recombination of enterovirus G strains in Japanese pigs: High prevalence of strains carrying a papain-like cysteine protease sequence in the enterovirus G population. *PLoS One* **2018**, *13*, e0190819.

108. Vidal, A.; Clilverd, H.; Cortey, M.; Martín-Valls, G.E.; Franzo, G.; Darwich, L.; Martín, M.; Mateu, E. Full-genome characterization by deep sequencing of rotavirus A isolates from outbreaks of neonatal diarrhoea in pigs in Spain. *Vet. Microbiol.* **2018**, *227*, 12–19.

109. Wang, H.; Li, S.; Mahmood, A.; Yang, S.; Wang, X.; Shen, Q.; Shan, T.; Deng, X.; Li, J.; Hua, X.; et al. Plasma virome of cattle from forest region revealed diverse small circular ssDNA viral genomes. *Virol. J.* **2018**, *15*, 11.

110. Wang, Y.; Zhang, W.; Liu, Z.; Fu, X.; Yuan, J.; Zhao, J.; Lin, Y.; Shen, Q.; Wang, X.; Deng, X.; et al. Full-length and defective enterovirus G genomes with distinct torovirus protease insertions are highly prevalent on a Chinese pig farm. *Arch. Virol.* **2018**, *163*, 2471–2476.

111. Wüthrich, D.; Boujon, C.L.; Truchet, L.; Selimovic-Hamza, S.; Oevermann, A.; Bouzalas, I.G.; Bruggmann, R.; Seuberlich, T. Exploring the virome of cattle with non-suppurative encephalitis of unknown etiology by metagenomics. *Virology* **2016**, *493*, 22–30.

112. Yang, C.; Wang, L.; Shen, H.; Zheng, Y.; Bade, S.A.; Gauger, P.C.; Chen, Q.; Zhang, J.; Guo, B.; Yoon, K.J.; et al. Detection and genetic characterization of porcine pegivirus in pigs in the United States. *Transbound. Emerg. Dis.* **2018**, *65*, 618–626.

113. Yang, L.E.; Zhao, Z.; Hou, G.; Zhang, C.; Liu, J.; Xu, L.; Li, W.; Tan, Z.; Tu, C.; He, B. Genomes and seroprevalence of severe fever with thrombocytopenia syndrome virus and Nairobi sheep disease virus in Haemaphysalis longicornis ticks and goats in Hubei, China. *Virology* **2019**, *529*, 234–245.

114. Yang, W. zhu; Yu, J. mei; Li, J. song; Cheng, W. xia; Huang, C. ping; Duan, Z. jun Genome characterization of a novel porcine bocavirus. *Arch. Virol.* **2012**, *157*, 2125–2132.

115. Yu, J.M.; Li, J.S.; Ao, Y.Y.; Duan, Z.J. Detection of novel viruses in porcine fecal samples from China. *Virol. J.* **2013**, *10*, 39.

116. Yu, X.; Jin, T.; Cui, Y.; Pu, X.; Li, J.; Xu, J.; Liu, G.; Jia, H.; Liu, D.; Song, S.; et al. Influenza H7N9 and H9N2 Viruses: Coexistence in Poultry Linked to Human H7N9 Infection and Genome Characteristics. *J. Virol.* **2014**, *88*, 3423–3431.

117. Zhang, B.; Tang, C.; Yue, H.; Ren, Y.; Song, Z. Viral metagenomics analysis demonstrates the diversity of viral flora in piglet diarrhoeic faeces in China. *J. Gen. Virol.* **2014**, *95*, 1603–1611.

118. Zhang, J.; Zheng, Y.; Xia, X.Q.; Chen, Q.; Bade, S.A.; Yoon, K.J.; Harmon, K.M.; Gauger, P.C.; Main, R.G.; Li, G. High-throughput whole genome sequencing of Porcine reproductive and respiratory syndrome virus from cell culture materials and clinical specimens using next-generation sequencing technology. *J. Vet. Diagnostic Investig.* **2017**, *29*, 41–50.

119. Zhao, L.; Niu, Y.; Lu, T.; Yin, H.; Zhang, Y.; Xu, L.; Wang, Y.; Chen, H. Metagenomic Analysis of the Jinding Duck Fecal Virome. *Curr. Microbiol.* **2018**, *75*, 658–665.

120. Zheng, S.; Wu, X.; Zhang, L.; Xin, C.; Liu, Y.; Shi, J.; Peng, Z.; Xu, S.; Fu, F.; Yu, J.; et al. The occurrence of porcine circovirus 3 without clinical infection signs in Shandong Province. *Transbound. Emerg. Dis.* **2017**, *64*, 1337–1341.
